# Supplementary figures and images for: BRM-SWI/SNF chromatin remodeling complex enables functional telomeres by promoting co-expression of TRF2 and TRF1
Source: PLoS Genet. 2020 Jun 5;16(6):e1008799. doi: 10.1371/journal.pgen.1008799 (PMC7299400; doi:10.1371/journal.pgen.1008799)

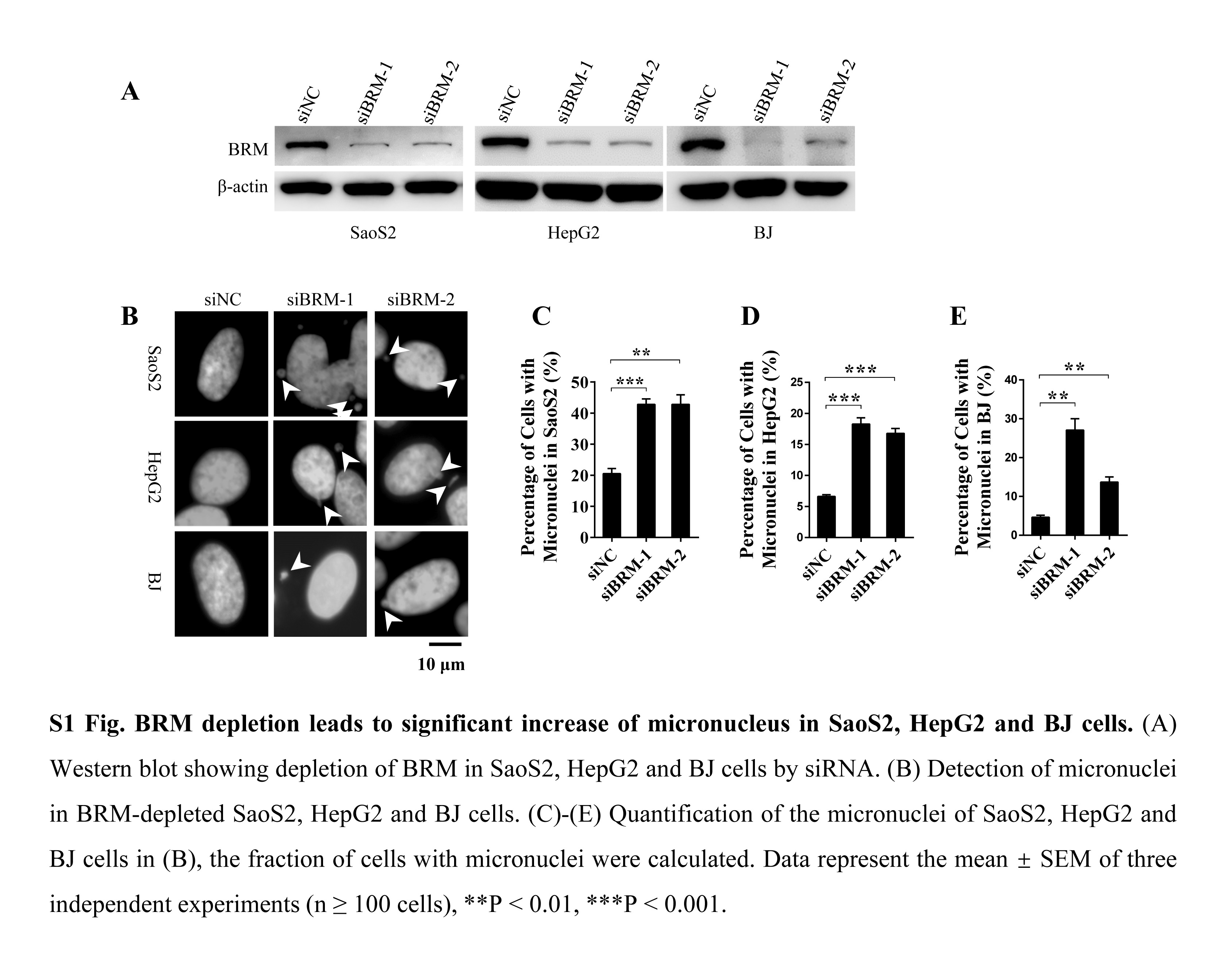

Supplement: S1 Fig — (A) Western blot showing depletion of BRM in SaoS2, HepG2 and BJ cells by siRNA. (B) Detection of micronuclei in BRM-depleted SaoS2, HepG2 and BJ cells. (C)-(E) Quantification of the micronuclei of SaoS2, HepG2 and BJ cells in (B), the fraction of cells with micronuclei were calculated. Data represent the mean ± SEM of three independent experiments (n ≥ 100 cells), **P < 0.01, ***P < 0.001. (TIF) [file pgen.1008799.s001.tif]

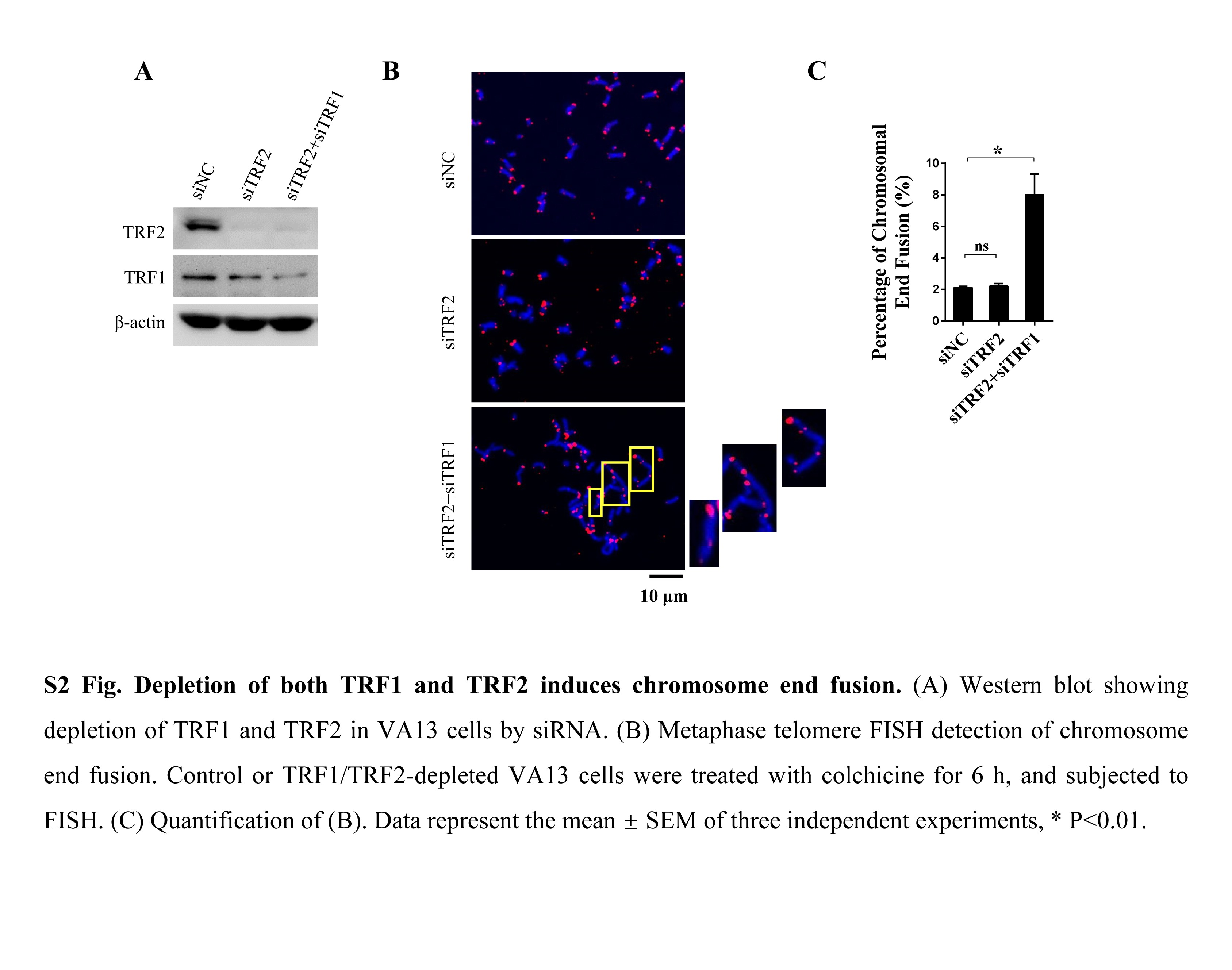

Supplement: S2 Fig — (A) Western blot showing depletion of TRF1 and TRF2 in VA13 cells by siRNA. (B) Metaphase telomere FISH detection of chromosome end fusion. Control or TRF1/TRF2-depleted VA13 cells were treated with colchicine for 6 h, and subjected to FISH. (C) Quantification of (B). Data represent the mean ± SEM of three independent experiments, *P<0.01. (TIF) [file pgen.1008799.s002.tif]

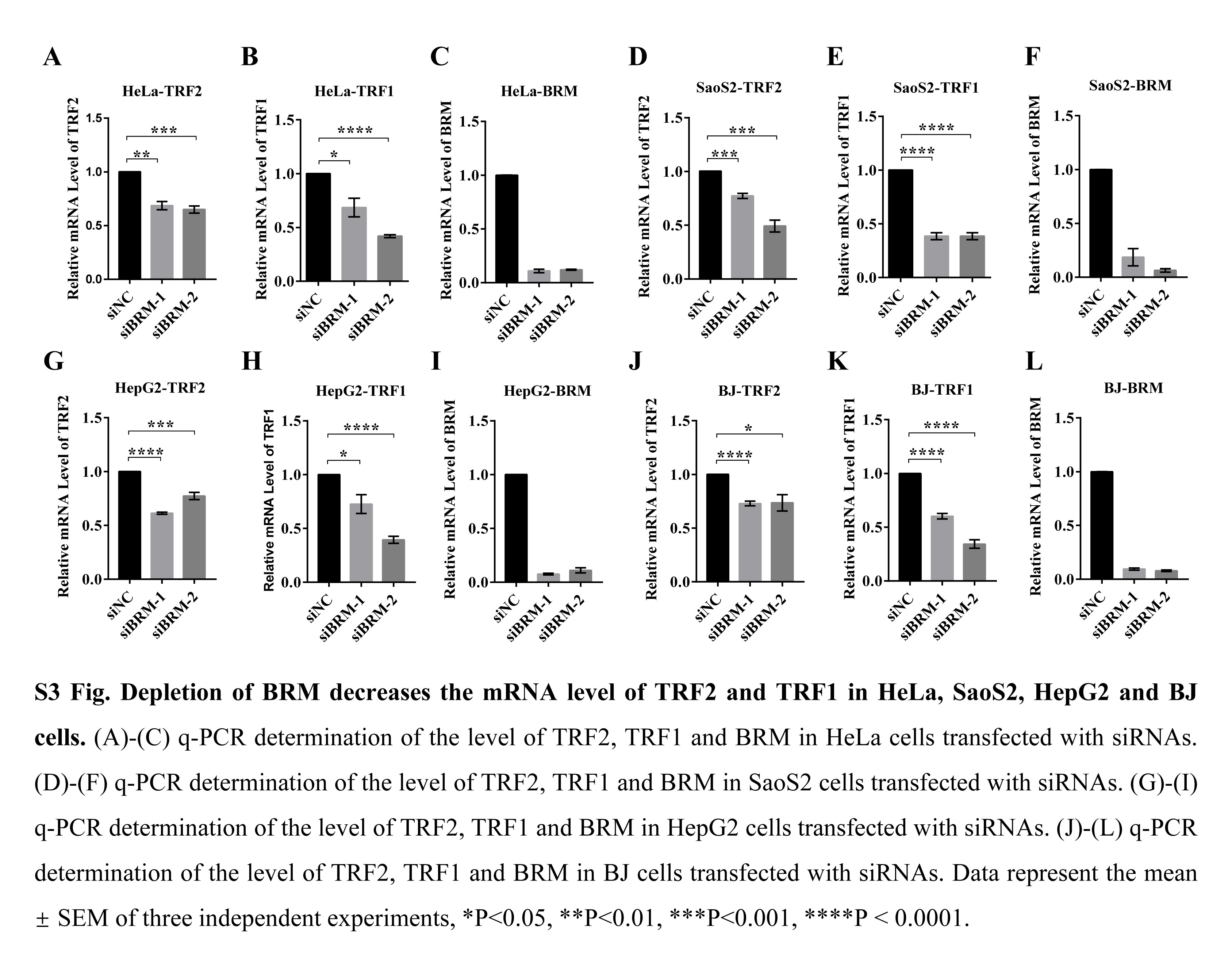

Supplement: S3 Fig — (A)-(C) q-PCR determination of the level of TRF2, TRF1 and BRM in HeLa cells transfected with siRNAs. (D)-(F) q-PCR determination of the level of TRF2, TRF1 and BRM in SaoS2 cells transfected with siRNAs. (G)-(I) q-PCR determination of the level of TRF2, TRF1 and BRM in HepG2 cells transfected with siRNAs. (J)-(L) q-PCR determination of the level of TRF2, TRF1 and BRM in BJ cells transfected with siRNAs. Data represent the mean ± SEM of three independent experiments, *P<0.05, **P <0.01, ***P<0.001, ****P < 0.0001. (TIF) [file pgen.1008799.s003.tif]

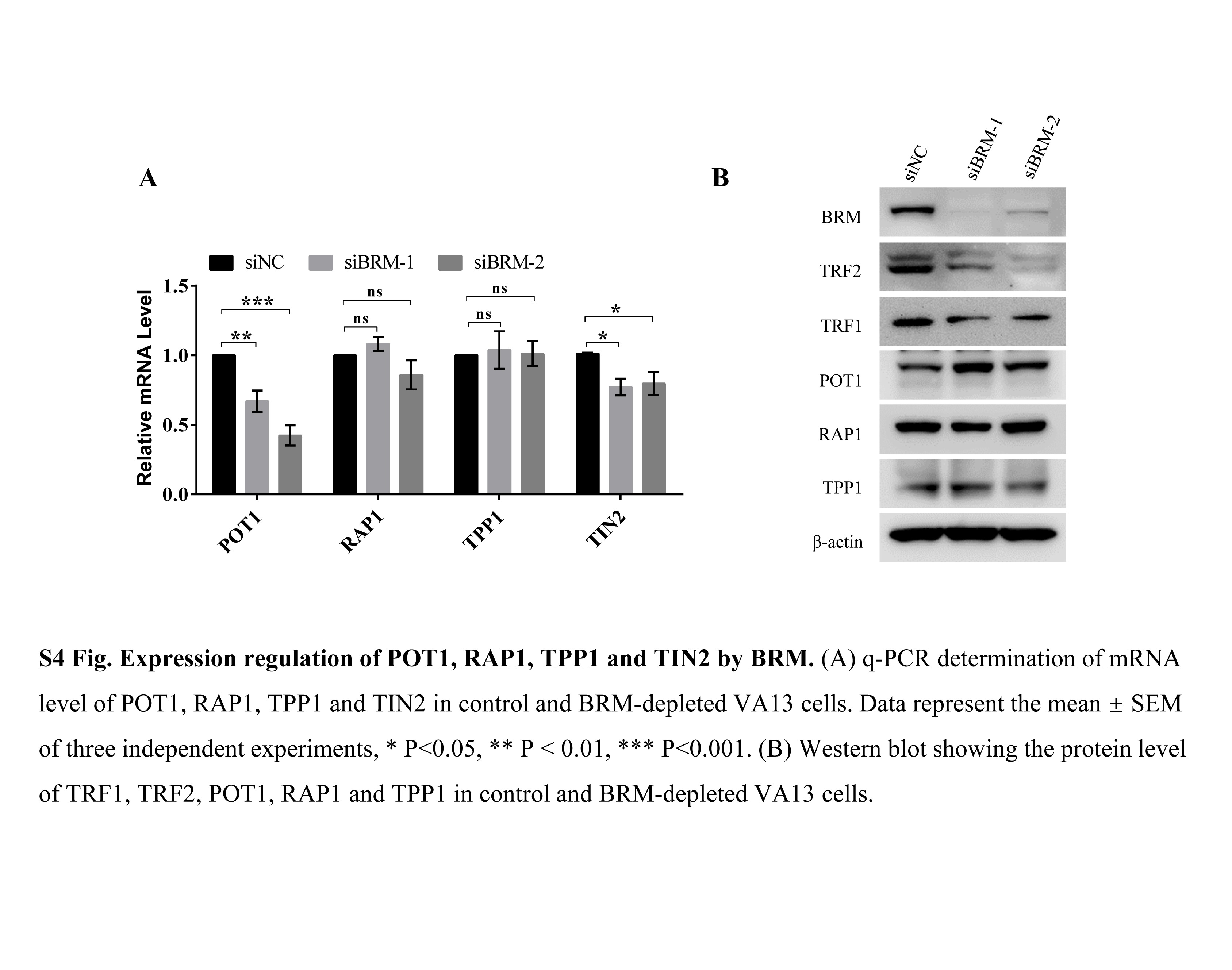

Supplement: S4 Fig — (A) q-PCR determination of mRNA level of POT1, RAP1, TPP1 and TIN2 in control and BRM-depleted VA13 cells. Data represent the mean ± SEM of three independent experiments, *P<0.05, **P < 0.01, ***P<0.001. (B) Western blot showing the protein level of TRF1, TRF2, POT1, RAP1 and TPP1 in control and BRM-depleted VA13 cells. (TIF) [file pgen.1008799.s004.tif]

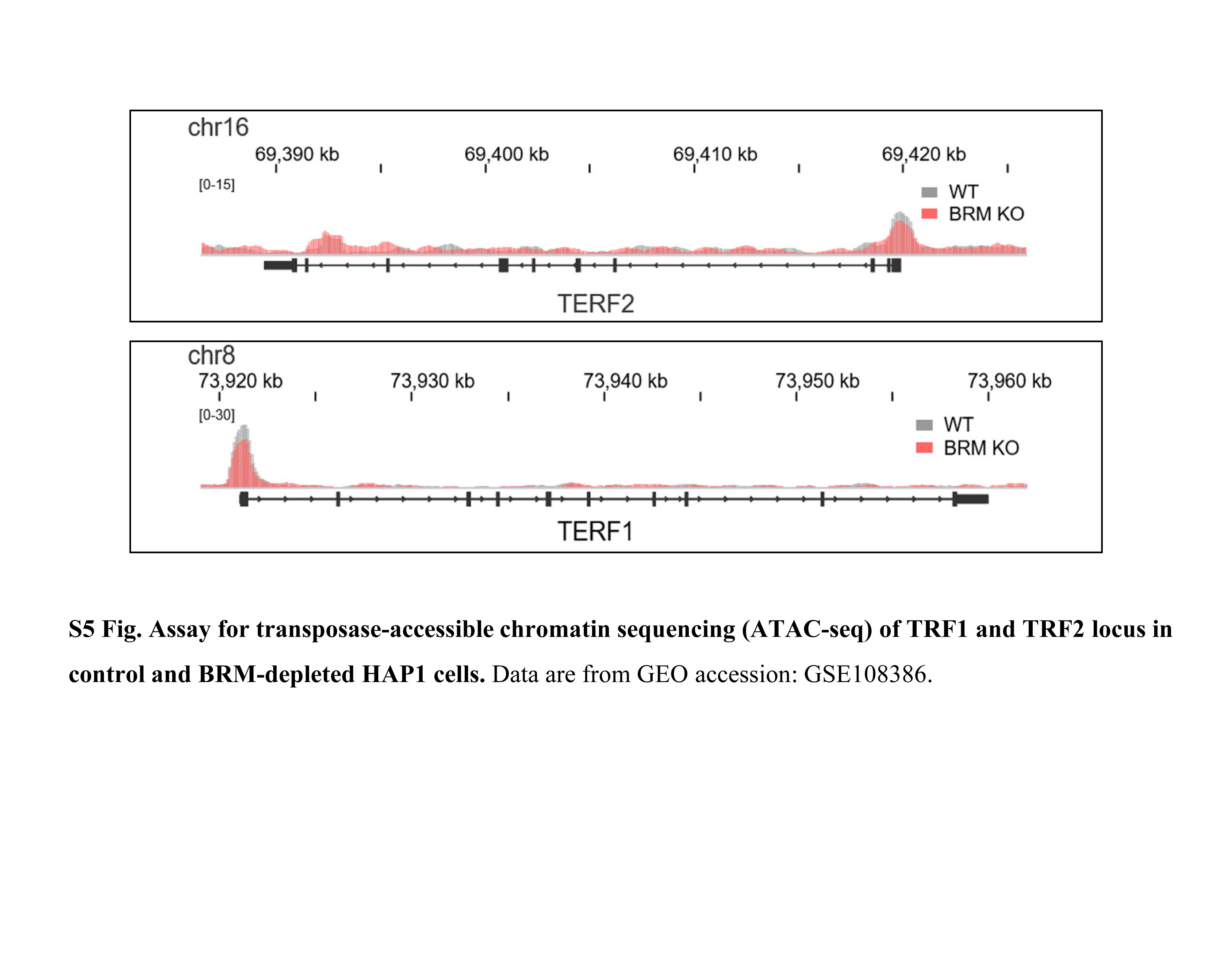

Supplement: S5 Fig — Data are from GEO accession: GSE108386. (TIF) [file pgen.1008799.s005.tif]

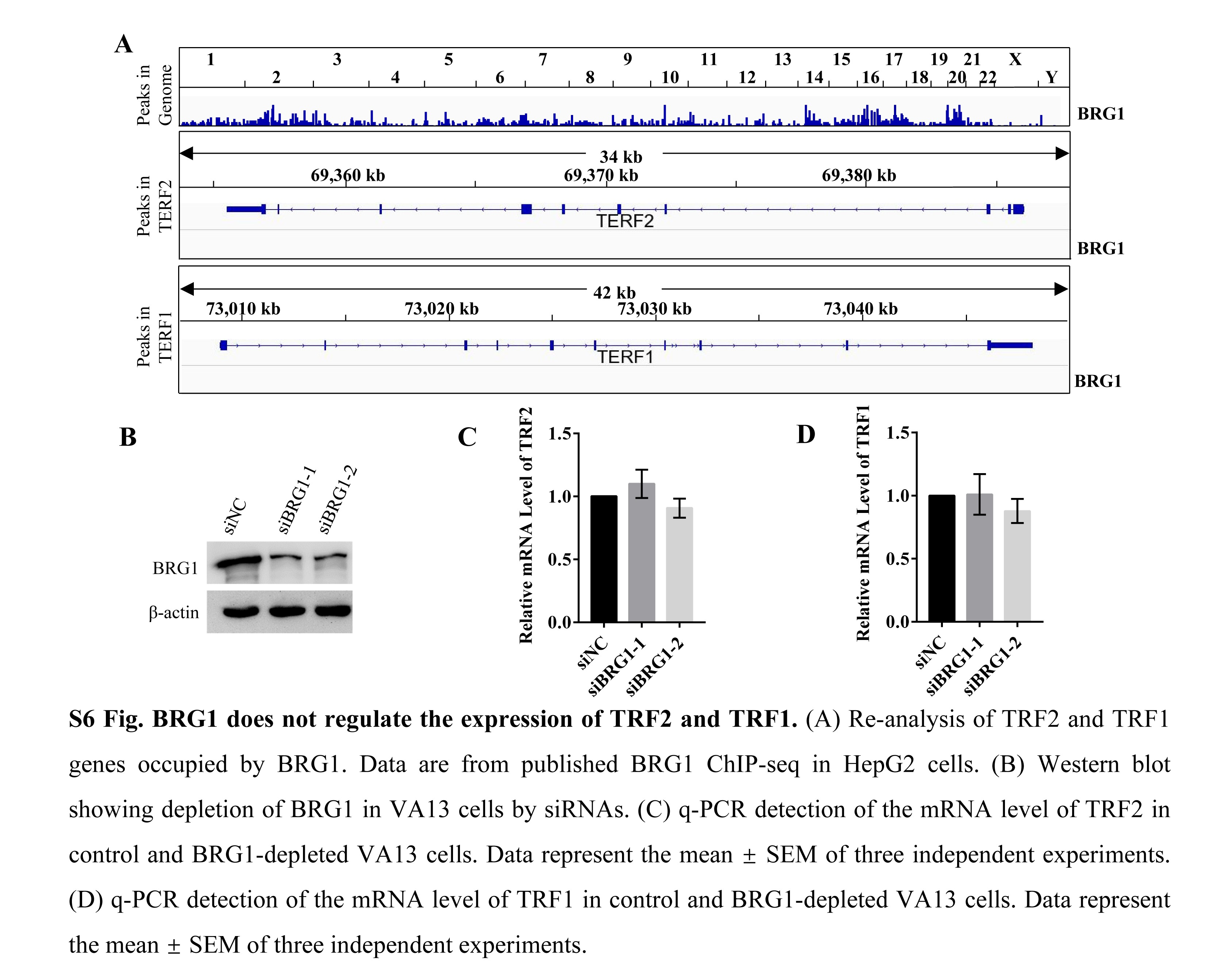

Supplement: S6 Fig — (A) Re-analysis of TRF2 and TRF1 genes occupied by BRG1. Data are from published BRG1 ChIP-seq in HepG2 cells. (B) Western blot showing depletion of BRG1 in VA13 cells by siRNAs. (C) q-PCR detection of the mRNA level of TRF2 in control and BRG1-depleted VA13 cells. Data represent the mean ± SEM of three independent experiments. (D) q-PCR detection of the mRNA level of TRF1 in control and BRG1-depleted VA13 cells. Data represent the mean ± SEM of three independent experiments. (TIF) [file pgen.1008799.s006.tif]
