# Supplementary material for: BRM-SWI/SNF chromatin remodeling complex enables functional telomeres by promoting co-expression of TRF2 and TRF1
Source: PLoS Genet. 2020 Jun 5;16(6):e1008799. doi: 10.1371/journal.pgen.1008799 (PMC7299400; doi:10.1371/journal.pgen.1008799)
Supplement: Attachment — Submitted filename: Response to reviewers comments.pdf [file pgen.1008799.s007.pdf]

Response to the reviewer's comments:

Reviewer #1: This manuscript described the molecular basis for how BRM-SWI/SNF complex contributes to telomere maintenance. The authors reported that RMB associates with TRF1 and TRF2 promoters and enhances their transcription. BRM knockdown led to reduced TRF1 and TRF2 expression and their occupancy at telomeres. In addition, BRM knockdown caused telomere uncapping, measured by gamma-H2AX foci formation at telomeres and chromosome end-to-end fusions; and telomere replication defects, evident by telomeric PCNA and RPA foci formation and fragile telomeres, which were mitigated by overexpressing TRF1 and TRF2. The work described in this manuscript is novel. The manuscript is well written. The experiments were well designed.

Major comments:

1. The authors demonstrated that some telomeres are TRF1 and TRF2 free in BRM knockdown cells by IF and FISH experiments. Did the authors quantify telomeric occupancy of TRF1 and TRF2 by ChIPs? Besides TRF1 and TRF2, did BRM deficiency affect other shelterin gene expression? Dr. de Lange laboratory showed that deletion in both TRF1 and TRF2 resulted in shelterin-free telomeres in mice. So Are TRF1- and TRF2-free telomeres bound by other shelterin proteins? It would be interesting to explore these questions in the follow-up studies, if these experiments are outside the scope of what could be accomplished within a timely revision period.

Response: As suggested, we performed ChIP experiment using TRF1 or TRF2 antibody to precipitate telomeric DNA in cells with or without BRM. The result showed that depletion of BRM leads to ~50% decrease of precipitated telomeric DNA for both TRF1 and TRF2 **(Fig. 4I and J) (Page 9, Line 173-176)**. This is consistent with ~40% increase of TRF1 or TRF2-free telomeres observed in BRM-depleted cells **(Fig. 4D and H)**.

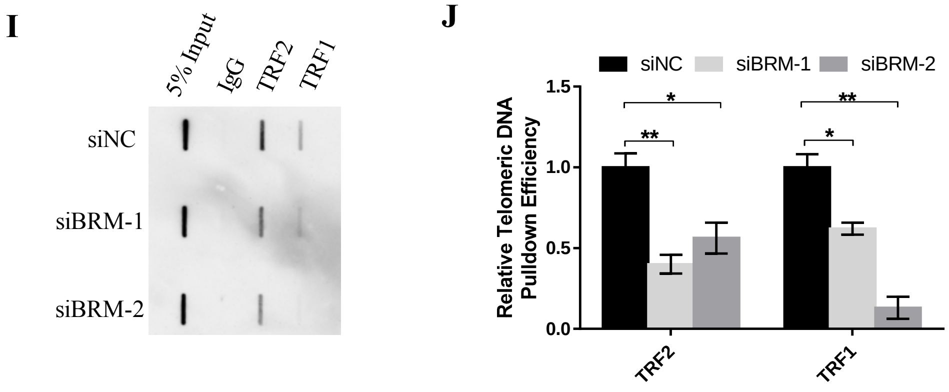

By q-PCR, we observed that knockdown of BRM results in significant decrease in POT1 mRNA, but limited or no change of RAP1, TPP1 and TIN2 transcripts. Western blot demonstrated no change in protein level of RAP1 and TPP1, but

increase of POT1 upon depletion of BRM. Inconsistent results between mRNA and protein level for POT1 may be due to translational and/or post-translational regulation of POT1 **(Fig. S4) (Page 10, Line 207-214)**.

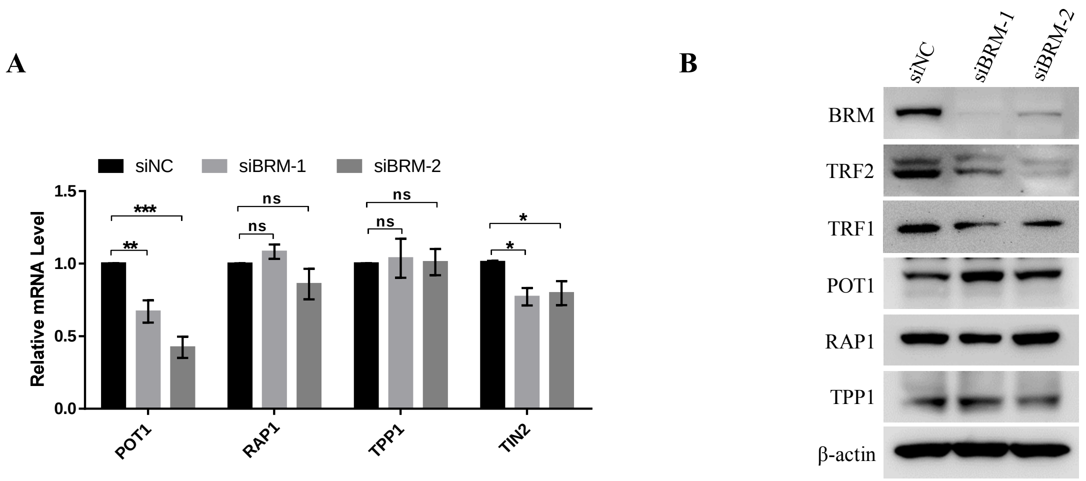

We agree with the reviewer that whether depletion of both TRF1 and TRF2 leads to shelterin free telomeres in human cells is very interesting question, which will be studied in the future. We thank the reviewer for these suggestions.

2. TRF1 and TRF2 overexpression could cause telomere and genome abnormalities, including rapid telomere loss, anaphase bridge, et al. Thus, it is important to show endogenous TRF1 and TRF2 expression in Figure 6A and B to justify TRF1 and TRF2 overexpression levels.

Response: In revised Figure 6A and B, both endogenous and overexpressed TRF1/TRF2 were shown by western blot using TRF1/TRF2 antibody. It appears that overexpressed TRF1/TRF2 are much greater in number than endogenous one. However, we don't observe significant increase of DNA damage response (DDR) or replication defect at telomeres when TRF2 or TRF1 is overexpressed (Fig. 6C-H). The easiest interpretation is that while superfluous TRF2 leading to replication defect was observed in telomerase positive cells (Nera et al., 2015), VA13 are used in our experiment, which are typical ALT cells bearing a high level of spontaneous DDR and replication stress that might make cells less sensitive or insensitive to replication defect induced by overexpressed TRF2 (Sobinoff and Pickett, 2017).

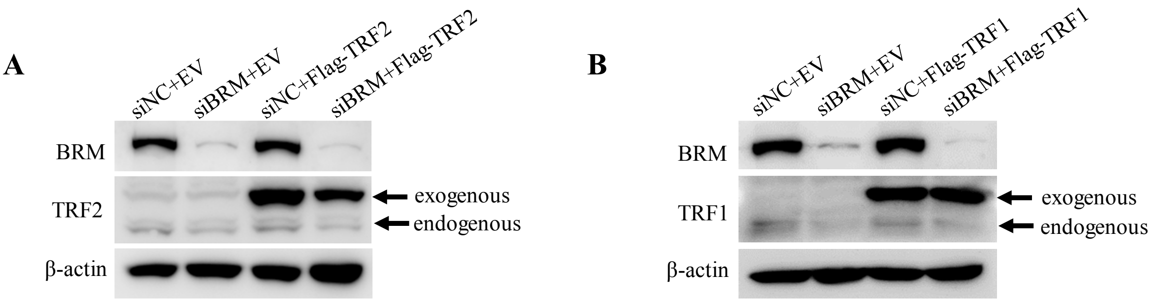

3. BRM knockdown cells showed increased end-to-end fusion. These cells had about 50% reduction in TRF2 levels (Figure 4). Because end-to-end fusion occurs when most TRF2 molecules are depleted in human cancer cell lines, I wonder are there other mechanisms contributing to the end-to-end fusion phenotype in BRM knockdown cells?

Response: We thank the reviewer for raising this very interesting question. We are also intrigued by this observation and thus performed the experiment to concurrently knockdown TRF2 and TRF1 using siRNAs. Surprisingly, we observed that while knockdown of TRF2 alone leads to no increase of chromosome fusion, knockdown of both TRF2 and TRF1 significantly increases the frequency of chromosome end fusion (From 2% to 8% in VA13 cells). This result implied that TRF1 may also participate in end protection by promoting the function of TRF2. Underlying mechanism is interesting topic which is worth studying in the future. We included this result in revised MS **(Fig. S2) (Page 9, Line 177-182)**.

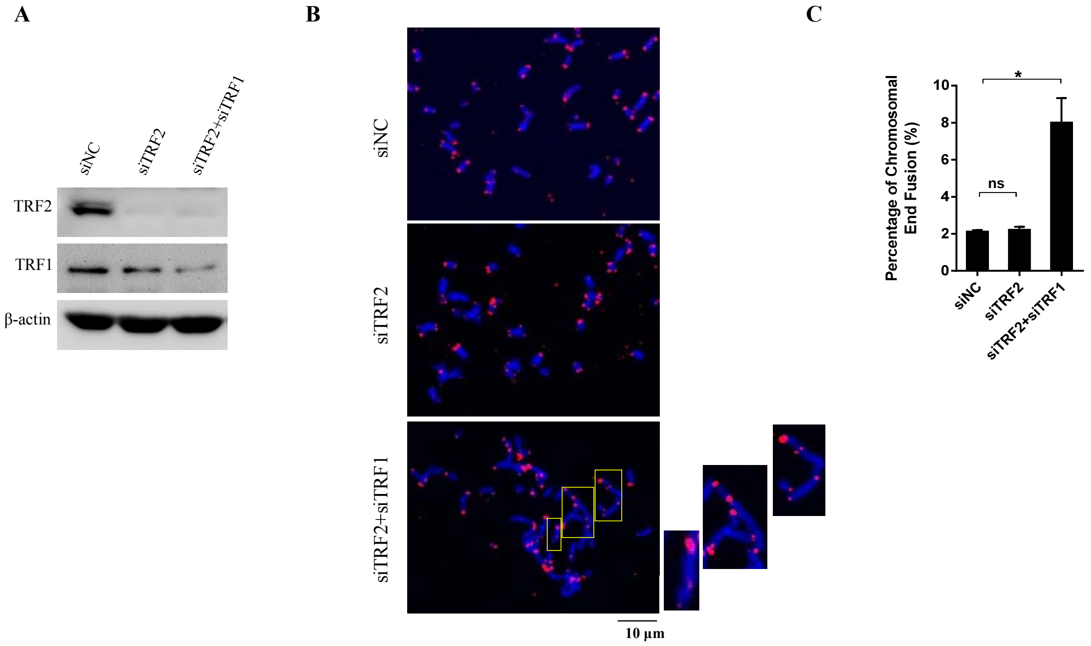

Minor comments:

Figure 5. TS should be TSS.

Response: We thank the reviewer for bringing this mistake to us. It has been corrected.

Page 5-6. "…BRM-SWI/SNF complex plays a role in chromatin end protection by telomeres…". "By telomeres" is confusing.

Response: It has been changed to "BRM-SWI/SNF complex plays a role in chromatin end protection is largely unknown". We thank the reviewer for pointing it out.

TIF analysis: Telomere signals are poor. How the authors define gamma-H2AX at telomeric site?

Response: The high-quality images were provided that clearly show the colocalization of gamma-H2AX and telomeres.

References

Sobinoff, A.P., and Pickett, H.A. (2017). Alternative Lengthening of Telomeres: DNA Repair Pathways Converge. Trends in genetics : TIG *33*, 921-932.

Nera, B., Huang, H.S., Lai, T., and Xu, L. (2015). Elevated levels of TRF2 induce telomeric ultrafine anaphase bridges and rapid telomere deletions. Nat Commun 6, 10132.

Reviewer #2: In this manuscript the authors define an essential role for BRM in maintaining telomere function by regulating the expression of TRF1 and TRF2. The experiments are logical and well-done, and the results are convincing that this mechanism is relevant in the two cell lines tested. The major concern is the generality of the phenotype across all cell types. While the correlation between BRM and TRF1 and TRF2 across cell lines is compelling, testing the dependency in additional cell lines would be essential to really support the conclusion. Testing BRM knockdown across a panel of cell lines for a dependency for TRF1/TRF2 transcriptional regulation along with at least one of the assays for genome stability would be sufficient.

Response: We thank the reviewer for their positive feedback and thoughtful suggestions. To confirm the generality of our findings, BRM regulating the expression of TRF1 and TRF2 has been examined in additional three cell lines (Saos2, HepG2, BJ fibroblast). We found that depletion of BRM significantly decreases mRNA level of TRF1 and TRF2 in all cell lines tested **(Fig. S3) (Page 10, Line 202-206).**

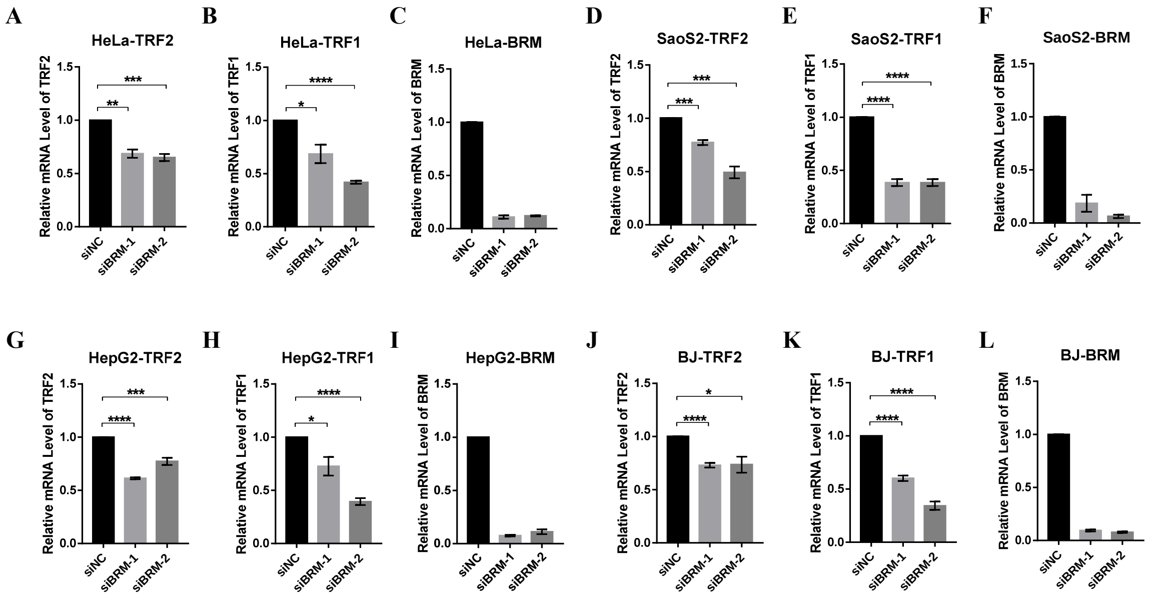

In addition, to determine the genome instability induced by deficient BRM, micronuclei assay was also performed on Saos2, HepG2 and BJ cell lines. Consistent with the observation in HeLa and VA13 cells, depletion of BRM leads to significant increase of micronuclei in these three cell lines (**Fig. S1**) **(Page 6, Line 116-117)**.

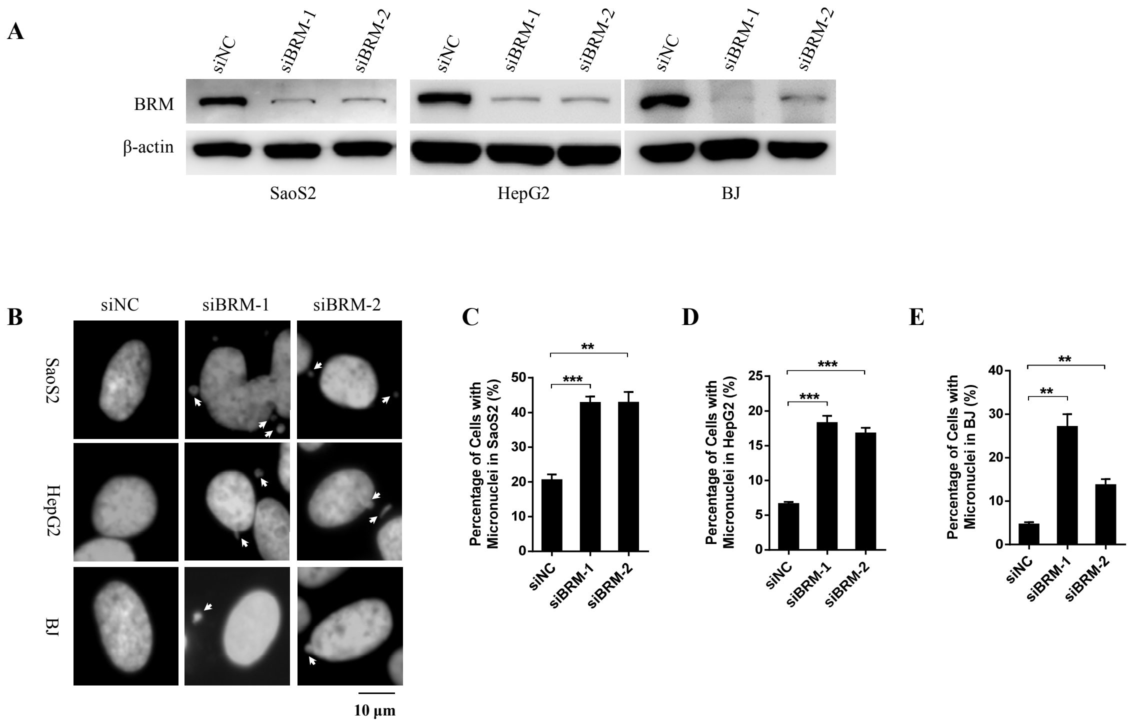

Is this a unique function for BRM that cannot be performed by BRG1? While it is clear that these two paralogs have functional differences, the ChIP-Seq data from HepG2 cells used in this manuscript (Raab et al 2017) indicates that BRM sites are also bound by BRG1 and that knockdown of each subunit can (but not necessarily) regulate genes in a similar manner. Does BRG1 knockdown similarly affect TRF1 and TRF2 expression or is this function exclusive to BRM? Based on a quick search, BRG1 expression displays the same correlation with TRF2 or TRF1 in the GTEx database. Why focus on BRM and not BRG1 for this study?

Response: We have determined whether depletion of BRG1 affects the expression of TRF1 and TRF2. It turns out that knockdown of BRG1 by two siRNA does not significantly change the expression level of TRF1 and TRF2. We also asked if BRG1 is localized to TRF1 or TRF2 gene. BRG1-occupied sites on genome was checked by analyzing BRG1 ChIP-seq data (Raab et al., 2017) . We found that there are no BRG1-occupied peaks at the region of TRF2 and TRF1 promoter or gene region. Therefore, transcriptional regulation of TRF1 and TRF2 is a unique function of BRM. The results were shown in **Fig. S6 (Page 13-14, Line 276-282)**.

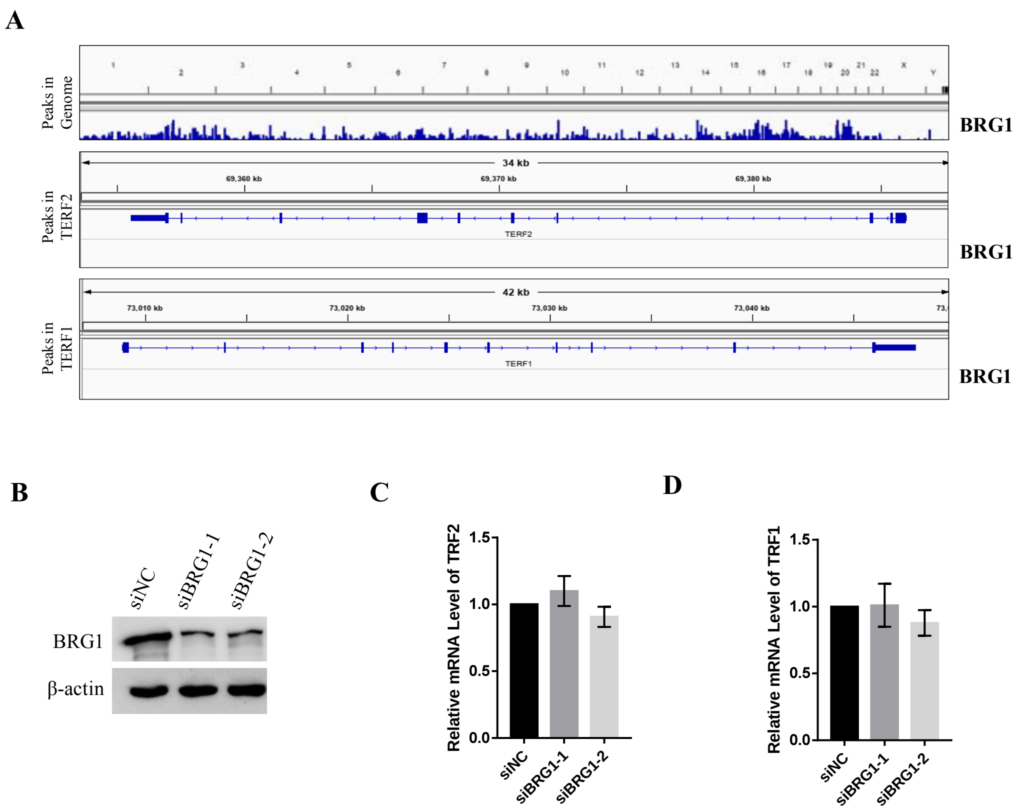

In a related issue, the BRM knockout mouse is reported to be phenotypically normal (although issues with whether or not these mice are actually knockouts have been raised). Could this mean that telomere dysfunction would only be observed upon acute depletion and that BRG1 can compensate in a long-term knockout setting? Can BRM knockout cells be cultured long term? Since many groups are trying to develop ways to specifically target BRM in BRG1 mutant lung cancers, understanding long term ramifications of BRM deletion in cells expressing both BRG1 and BRM is particularly important.

Response: When BRM is stably knocked down in VA13 cells using shRNA, cells underwent apoptosis. But, a small portion of cells were able to survive and started to proliferate after about two weeks (See the proliferation curve below). We then examined the expression of BRM, TRF1 and TRF2 in survived cells and found that while BRM is still suppressed by shRNA, the expression of TRF1 and TRF2 are largely rescued. We speculated that BRG1 may compensate the loss of BRM in survived cells. To test it, we knocked down BRG1 in these cells and determined the expression level of BRM, TRF1 and TRF2. Different from the expectation that BRG1 deficiency would lead to decreased TRF1 and TRF2, we observed that depletion of BRG1 slightly increases mRNA level of BRM, TRF1 and TRF2. Because level of BRM is still much lower than control cells and thus is not sufficient to rescue TRF1 and TRF2, we hypothesized that other unknown factors, but not BRG1, may function to rescue the expression of TRF1 and TRF2 in BRM-depleted cells.

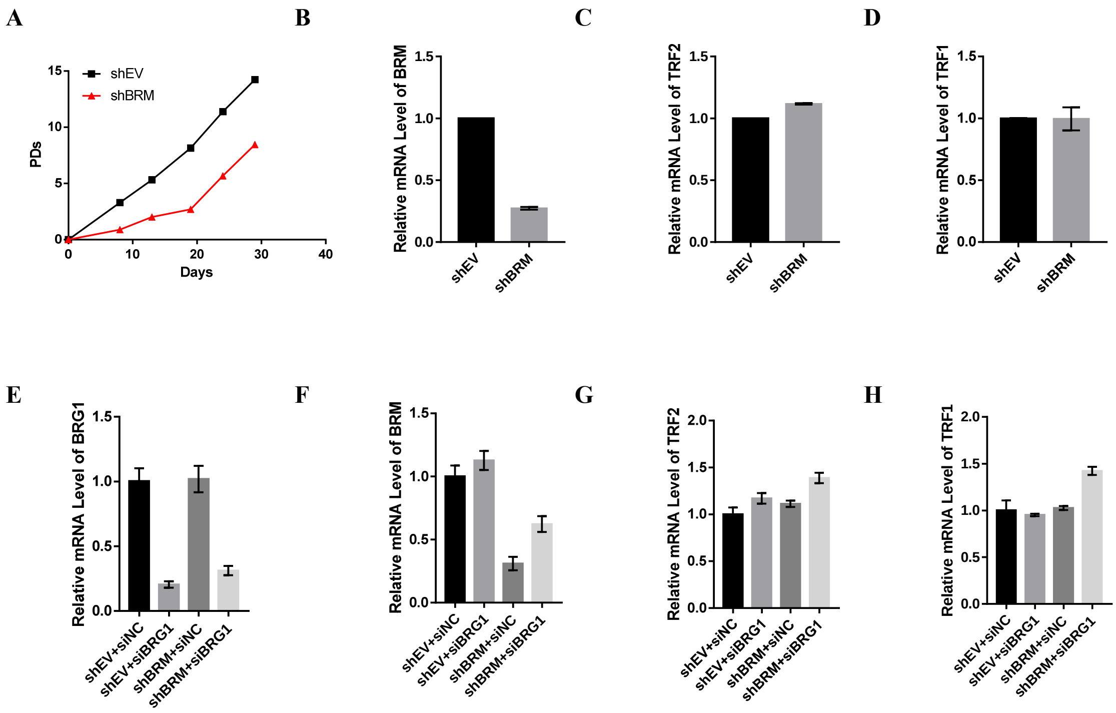

References

Raab, J.R., Runge, J.S., Spear, C.C., and Magnuson, T. (2017). Co-regulation of transcription by BRG1 and BRM, two mutually exclusive SWI/SNF ATPase subunits. Epigenetics & chromatin *10*, 62.

Reviewer #3: Title: BRM-SWI/SNF Chromatin Remodeling Complex Enables Functional Telomeres by Promoting Co-expression of TRF2 and TRF 1

This paper shows that BRM-SWI/SNF Chromatin Remodeling Complex is important for maintaining telomere function by promoting expression of TRF1 and TRF2. BRM-SWI/SNF complex remodels chromatin and binds to TRF2 and TRF1 promoters to regulate their expression. In contrast, depletion of BRM decreases TRF2 and TRF1 transcripts. The findings are novel and interesting to the field of telomere biology. Excitingly, BRM depletion-induced genome instability is mainly caused by telomere dysfunction due to the lacking of TRF2 and TRF1, and exogenous expression of TRF2 and TRF1 completely rescues genome instability and cell apoptosis. Previously, the authors revealed that BRG1-SWI/SNF chromatin remodeling complex is engaged in telomere length maintenance by regulating hTERT expression (15). Does BRG1 SWI/SNF also regulates expression of TERT or TRF1/2? Is BRG1 or BRM function in telomere cell type specific?

Response: We explored how depletion of BRG1 affects the expression of TRF1 and TRF2. It turns out that knockdown of BRG1 does not significantly change the mRNA level of TRF1 and TRF2 (**Fig. S6B-D**) **(Page 13-14, Line 276-282)**.

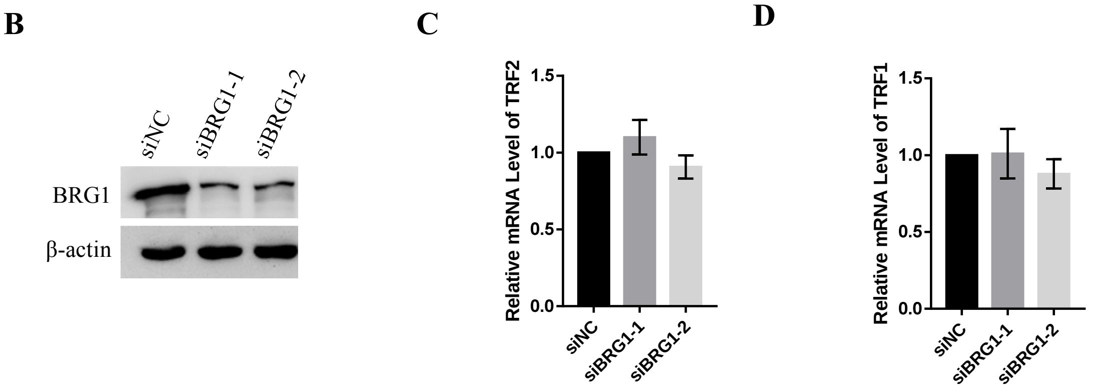

To confirm the generality of our findings, BRM regulating the expression of TRF1 and TRF2 has been examined in additional three cell lines (Saos2, HepG2, BJ fibroblast). We found that depletion of BRM significantly decreases mRNA level of TRF1 and TRF2 in all cell lines tested **(Fig. S3) (Page 10, Line 202-206).**

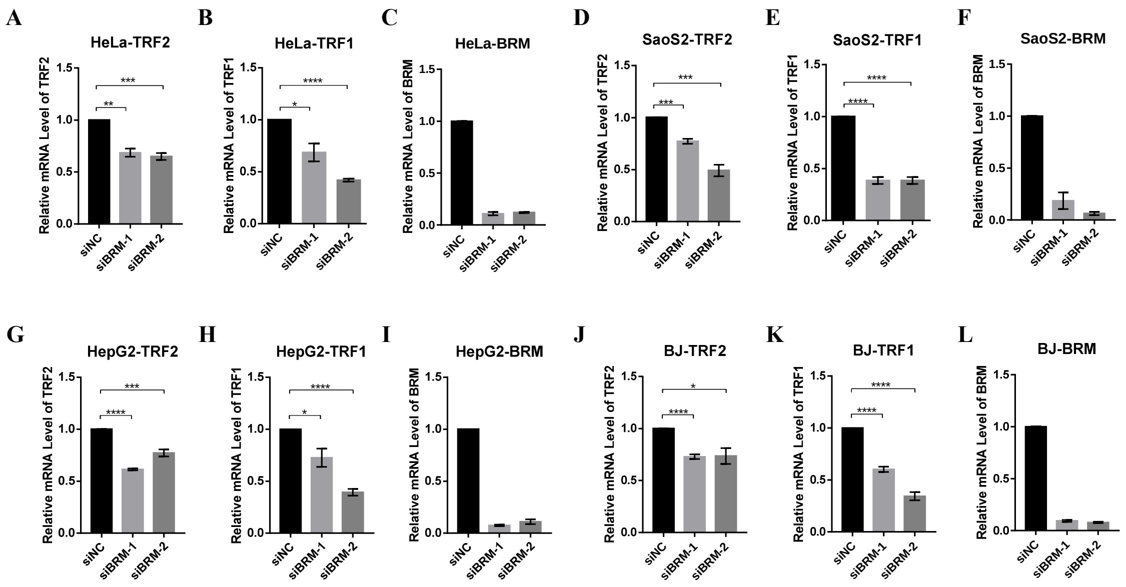

Specific comments:

1) VA13, HeLa, 293T were used for this study. Why these three cell lines were used? The reasons were not given.

Response: HeLa and VA13 are a typical telomerase positive and ALT (telomerase negative using ALT mechanism to maintain telomere length) cell line, respectively. 293T cells are used to produce the lentivirus due to their high transfection efficiency. This has been mentioned in revised MS **(Page 6, Line 109-111)**. We thank the reviewer for bringing this issue to us.

2) Chromatin remodeling complex and telomere regulation are important for embryonic stem cells, so embryonic stem cells could be good model studying the function of BRM-SWI/SNF Chromatin Remodeling Complex and TRF1 and TRF2. Does BRM-SWI/SNF regulate TRF2 and TRF1 in embryonic stem cells? or discussed?

Response: It is certainly possible that BRM also regulates TRF1 and TRF2 expression in ES cells. To test it, we knocked down BRM by siRNA in human H1 ES cells and found that depletion of BRM leads to mass death of ES cells and there are no much cells left after 48h for further assay (See picture below). This phenotype was discussed in revised MS **(Page 13, Line 271-275)**.

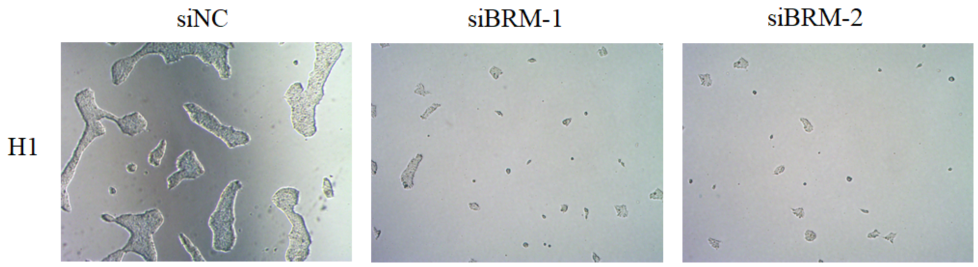

3) Re-analysis of public ChIP-seq data Published ChIP-seq data from HepG2 cells upon transfection with shNS (GEO accession: GSE102559) (23) was re-analyzed in this study. The biding of the protein by ChIP may differ among cell types. Can the ChIP biding data from HepG2 cells be applied to other three cell types used in this study?

Response: From HepG2 ChIP-seq (Raab et al., 2017), we found that TRF1 and TRF2 genes are occupied by BRM. This was immediately confirmed in VA13 cells by ChIP assay coupled with PCR, implying that BRM may regulate the expression of TRF1 and TRF2 **(Fig. 5B-E)**. Indeed, BRM depletion led to significant decrease of TRF1 and TRF2 expression in VA13, HeLa, HepG2, Saos2, and BJ **(Fig. 5H and I, Fig. S3)**. We thus concluded that BRM is a positive regulator for expression of TRF1 and TRF2.

4) The authors imply that BRM-SWI/SNF remodels chromatin and activates TRF2 and TRF1 expression. But no data is available to show that BRM-SWI/SNF open chromatin at TRF2 and TRF1 promoters or enhancers. It is unknown whether BRM-SWI/SNF opens chromatin at genome-wide. Perhaps ATAC may provide some answers.
Response: As suggested, we analyzed ATAC-seq profile of TRF1 and TRF2 locus in control and BRM-depleted HAP1 cells (GEO accession: GSE108386)(Schick et al., 2019). It was revealed that knockout of BRM leads to reduction of chromatin accessibility at promoter region of TRF1 and TRF2. This is consistent with the presumption that BRM-SWI/SNF chromatin remodeling complex could promotes gene transcription by decondensing associated chromatin. This has been discussed in revised MS **(Fig. S5) (Page 12, Line 254-257).** We thank the reviewer for their invaluable comments that strengthen our findings.

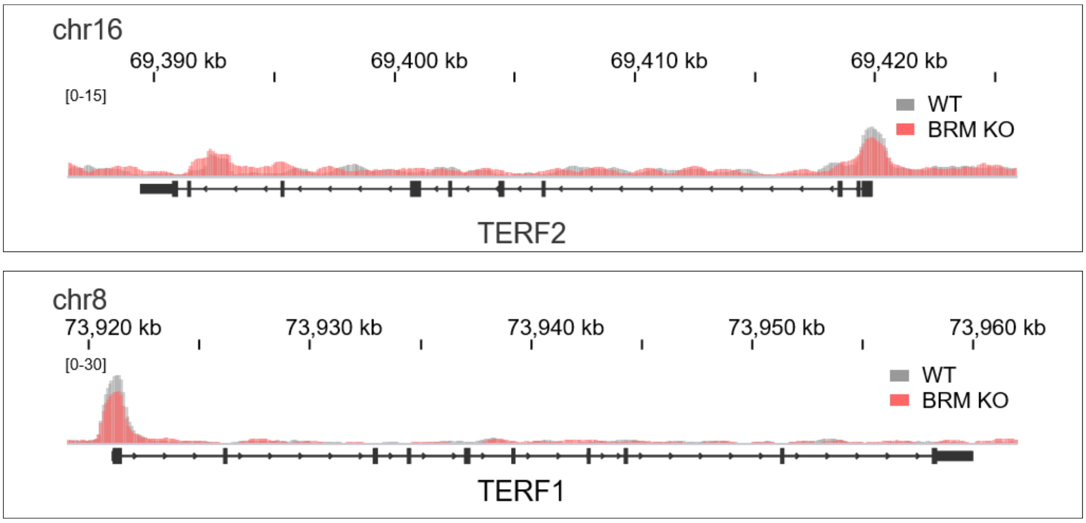

References

Raab, J.R., Runge, J.S., Spear, C.C., and Magnuson, T. (2017). Co-regulation of transcription by BRG1 and BRM, two mutually exclusive SWI/SNF ATPase subunits. Epigenetics & chromatin *10*, 62.

Schick, S., Rendeiro, A.F., Runggatscher, K., Ringler, A., Boidol, B., Hinkel, M., Májek, P., Vulliard, L., Penz, T., Parapatics, K., *et al.* (2019). Systematic characterization of BAF mutations provides insights into intracomplex synthetic lethalities in human cancers. Nature genetics *51*, 1399-1410.
